# The Heirloom

**Published:** 1879-09

**Authors:** 


THE HEIRLOOM of a family is literally the apparatus for weaving cloth. In early history it was an indispensable article of household furniture, was well taken care of, and lasted for generations; was kept in affectionate memory of those who had gone before. In Germany and Holland the petticoat was considered the heirloom, worn by mother and daughter, and grand-daughter sometimes; so well were they made, and of so substantial a material, and so cleanly kept, that they seemed almost to never wear out.

